# Methodological confounds of measuring urinary oxidative stress in wild animals

**DOI:** 10.1002/ece3.9115

**Published:** 2022-07-17

**Authors:** Zoe E. Melvin, Hussein Dhirani, Christopher Mitchell, Tim R. B. Davenport, Jonathan D. Blount, Alexander V. Georgiev

**Affiliations:** ^1^ School of Natural Sciences, Bangor University Bangor UK; ^2^ Zanzibar Red Colobus Project Bangor University Bangor UK; ^3^ Centre for Ecology & Conservation, College of Life & Environmental Sciences University of Exeter, Penryn Campus Penryn UK; ^4^ ReWild Arusha Tanzania

**Keywords:** 8‐oxodG, DNA damage, ecophysiology, lipid peroxidation, non‐invasive sampling, oxidative stress, Zanzibar red colobus

## Abstract

Biomarkers of oxidative stress (OS) are useful in addressing a wide range of research questions, but thus far, they have had limited application to wild mammal populations due to a reliance on blood or tissue sampling. A shift toward non‐invasive measurement of OS would allow field ecologists and conservationists to apply this method more readily. However, the impact of methodological confounds on urinary OS measurement under field conditions has never been explicitly investigated. We combined a cross‐sectional analysis with a field experiment to assess the impact of four potential methodological confounds on OS measurements: (1) time of sampling, (2) environmental contamination from foliage; (3) delay between sample collection and flash‐freezing in liquid nitrogen; and (4) sample storage of up to 15 months below −80°C. We measured DNA oxidative damage (8‐hydroxy‐2′‐deoxyguanosine, 8‐OHdG), lipid peroxidation (malondialdehyde, MDA), total antioxidant capacity (TAC), and uric acid (UA) in 167 urine samples collected from wild Zanzibar red colobus (*Piliocolobus kirkii*). We found that MDA was higher in samples collected in the morning than in the afternoon but there were no diurnal patterns in any of the other markers. Contamination of samples from foliage and length of time frozen at −80°C for up to 15 months did not affect OS marker concentrations. Freezing delay did not affect OS levels cross‐sectionally, but OS values from individual samples showed only moderate‐to‐good consistency and substantial rank‐order reversals when exposed to different freezing delays. We recommend that diurnal patterns of OS markers and the impact of storage time before and after freezing on OS marker concentrations be considered when designing sampling protocols. However, given the high stability we observed for four OS markers subject to a variety of putative methodological confounds, we suggest that urinary OS markers provide a valuable addition to the toolkit of field ecologists and conservationists within reasonable methodological constraints.

## Graphical abstract

Urinary biomarkers of oxidative stress (OS) would allow field ecologists and conservationists to address a wealth of new questions. However, we must ensure that OS measurements from field studies are robust and reliable. We recommend that diurnal patterns of OS markers and the impact of storage time before and after freezing on OS marker concentrations be considered when designing sampling protocols, however, in general, urinary OS markers provide a valuable addition to the toolkit of field researchers within reasonable methodological constraints.

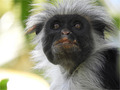

## 1 INTRODUCTION

Non‐invasive techniques have revolutionized the field of ecophysiology by, firstly, reducing the adverse effects of field research on animal welfare and behavior, and, secondly, limiting the confounding effects of the stress of capture and restraint on biomarker measurements (Costantini et al., 2017; Nwunuji et al., 2014). This has allowed us to address new questions in previously inaccessible study systems (Behringer & Deschner, 2017; Narayan, 2013). But while non‐invasive methods are commonly applied to study animal energetics (Emery Thompson, 2017), endocrinology (McCormick & Romero, 2017) and, more recently, immune function (Behringer et al., 2017; Tombak et al., 2020), non‐invasive studies of oxidative stress physiology in the wild are rare (Thompson González et al., 2020).

Oxidative stress (OS) is caused by the imbalance between the production of reactive oxygen species and the production, dietary intake, and repairing action of antioxidants. Oxidative stress can damage cellular DNA, proteins, and lipids (Valko et al., 2007). This can have negative consequences for health, reproduction, and survival (Bize et al., 2008; Saino et al., 2011; Sebastiano et al., 2017) making OS markers highly relevant to individual fitness. Additionally, oxidative stress is universal to aerobic organisms, meaning that OS can be measured in a wide range of taxa (Beaulieu & Costantini, 2014). Uniquely among physiological markers, both the damage and protection aspects of OS can be measured separately, meaning that OS markers can give us unparalleled insights into both the costs facing an organism and an organism's ability to cope with these costs (Beaulieu & Costantini, 2014). These properties of OS markers make them particularly suitable tools for the study of life‐history trade‐offs (Blount et al., 2016; Monaghan et al., 2009; Speakman et al., 2015; Thompson González et al., 2020) and the impacts of anthropogenic disturbance (Semeniuk et al., 2009). Indeed, many studies have investigated these topics in wild animals but have relied exclusively on blood or tissue samples to quantify OS (e.g., *Ovis aries*: Nussey et al., 2009; Christensen et al., 2016; *Mungos mungo*: Vitikainen et al., 2016; *Macaca mulatta*: Georgiev, Muehlenbein, et al., 2015; Georgiev, Emery Thompson, et al., 2015; *Dasyatis americana*: Semeniuk et al., 2009; and *Mandrillus sphinx*: Beaulieu et al., 2014). The reliance on invasive sampling has thus precluded the broader adoption of OS markers in field research, especially in the case of large, endangered, and difficult‐to‐capture animals.

A shift to a non‐invasive approach for quantifying OS will allow us to study a wider range of animals, address research questions requiring longitudinal measurements, and reduce the risk posed to study species. Urinary OS markers provide a promising alternative to blood and tissue sampling for field studies. Markers of OS are more stable in urine than in blood (Il'yasova et al., 2012) and have been used in clinical research since at least the 1980s (Cathcart et al., 1984). More recently, urinary OS markers have been applied in studies of captive animals in laboratory and zoo settings (Cho et al., 2009; Marchal et al., 2013; Costantini et al., 2021,) but thus far, only one study has measured OS in wild animals via non‐invasive sampling (Thompson González et al., 2020). In their study of wild chimpanzees, Thompson González et al. (2020) showed that MDA‐TBARS (a marker of lipid peroxidation) was higher and total antioxidant capacity was lower later in the day and they found a weak negative relationship between storage time and MDA‐TBARS. However, a more explicit investigation of a broader range of methodological confounds in measuring OS in the field is essential to aid the planning of robust field research using OS markers in the future.

In this study we, therefore, aimed to explicitly examine four major confounds that can affect the interpretation of OS values in opportunistically collected urine samples in a remote field setting: (1) time of sampling; (2) environmental contamination from foliage during sample collection; (3) a delay between sample collection and flash‐freezing in liquid nitrogen; and (4) prolonged sample storage at or below −80°C.

First, any circadian variation in marker excretion can be problematic when relying on imbalanced datasets that are characteristic of opportunistic sampling. Evidence of diurnal variation in OS is mixed and differs between markers and sample media, both in humans (Alajbeg et al., 2017; Singh et al., 2004; Valencia et al., 2001) and chimpanzees (Thompson González et al., 2020). Therefore, an assessment of diurnal variation in multiple OS markers in an additional species, in particular a non‐ape species, will provide a valuable addition to this literature and aid in determining the potential impact of imbalanced sampling on the analysis of OS markers.

Second, we also examined the effect of possible environmental contamination on OS marker levels. Evidence for significant and consistent impacts of environmental contaminants on urinary biomarkers is mixed, therefore a marker‐specific assessment of such confounds is usually necessary (Braga Goncalves et al., 2016; Heistermann & Higham, 2015; Higham et al., 2020; Schwartz & Granger, 2004). Environmental contamination of samples can be introduced through a variety of ways, for example, mixing with soil, feces, or contact with vegetation. Contamination of samples through contact with vegetation is one of the most difficult sources of contamination to avoid because the collection of urine directly from the stream of a wild animal is not always possible and samples are regularly collected from the surface of vegetation (Fedurek et al., 2016; Higham et al., 2011; Rincon et al., 2019; Surbeck et al., 2012; Thompson González et al., 2020). Although the potential effects of leaf‐borne contaminants on urinary biomarker measurement have been successfully ruled out for some metabolites (testosterone and creatinine: Muller & Wrangham, 2004; Marshall & Hohmann, 2005; and estrone conjugates: Knott, 2005), this is yet to be confirmed for OS markers.

Third, we assessed the effect of a delay between sample collection and flash‐freezing in liquid nitrogen on OS measurements, something which is often unavoidable and difficult to standardize at remote field sites. Markers of oxidative status are generally considered stable during short‐term storage before freezing (blood: at 0–4°C for 40 h and at 21–22°C for 40 h, Koracevic et al., 2001; 4°C for up to 24 h, Nussey et al., 2009; 3 h to 48 h at 4°C and 20°C, Jansen et al., 2013a; urine: 20°C for 26 h, Lee & Kang, 2008; 4°C and 25°C for 24 h, Matsumoto et al., 2008). While these results are promising, whether the same degree of urinary marker stability would be retained at the higher ambient temperatures often found in field conditions in the tropics requires evaluation.

Fourth, we also considered how duration of frozen storage affects OS markers. Field studies of wild animals are often conducted in remote locations with limited access to laboratory equipment meaning samples are often stored for months or years before analysis. Additionally, length of time in storage is a particularly difficult confound to standardize because samples are normally collected over a long period of time and are assayed in the lab in one or several batches. Therefore, ensuring stability of OS markers during storage is necessary to reliably compare samples. OS markers in blood have been found to be stable in long‐term storage after freezing for up to 2 years (−20°C for 1 month, Koracevic et al., 2001; −20°C, −80°C and − 196°C for 12 months, Jansen et al., 2013b; −80°C for 60 months, Jansen et al., 2017). However, the long‐term stability of OS markers in urine is less certain and seems to vary by marker. Urinary MDA levels have been shown to decline over long‐term frozen storage (30 days at −20°C, Martinez & Kannan, 2018; 1–10 years at −30°C, Thompson González et al., 2020) while other markers remained stable (800 days at −80°C, Matsumoto et al., 2008; 30 days at −20°C, Martinez & Kannan, 2018; and 1–10 years at −30°C, Thompson González et al., 2020). The extent to which such declines would be observed in other urinary OS markers and the timeline of such effects require further study.

We investigated the effect of these four putative methodological confounds on OS marker levels in the Zanzibar red colobus (*Piliocolobus kirkii*), an endangered primate for which no physiological data have been reported either from the wild or from captivity. The redox status of an organism is the result of a complex cascade of processes and therefore it is necessary to measure multiple markers representing different aspects of these processes to properly capture the OS an animal is facing (Speakman et al., 2015). We chose four complementary OS markers representing different aspects of the oxidative status of the animal; two markers of oxidative damage: 8‐hydroxy‐2′‐deoxyguanosine (8‐OHdG), a marker of DNA oxidative damage and malondialdehyde (MDA), a marker of lipid peroxidation, and two markers of antioxidant capacity: total antioxidant capacity (TAC) and uric acid (UA). These markers are known to be stable, represent system‐wide levels of OS, and have a variety of commercial assays available to test for them. Because of this, these markers have been used to measure OS in a variety of contexts, for example, in studies of wildlife conservation (French et al., 2017), life history (Christensen et al., 2016; Thompson González et al., 2020), behavioral ecology (Beaulieu et al., 2014; Georgiev, Emery Thompson, et al., 2015; Georgiev, Muehlenbein, et al., 2015), and socioecology (Costantini et al., 2017). Therefore, our choice of markers represents a useful marker set for ecologists and conservationists.

We examined how the concentrations of these four markers were affected by (1) time of day; (2) environmental contamination from leaf surfaces; (3) sample freezing delays (time between sample collection and flash‐freezing in liquid nitrogen); and (4) time elapsed between freezing and laboratory analysis. We did not have clear expectations regarding the presence of diurnal variation in marker values given the lack of consistent patterns in previous studies nor did we have predictions about the direction of the effect of environmental contamination on marker concentrations given that there is no previous research on this topic. We expected that longer freezing delays and longer time spent frozen would be linked to decreased levels of all OS markers because these markers are expected to degrade over time.

## 2 MATERIALS AND METHODS

### 2.1 Study site and subjects

We sampled 40 wild Zanzibar red colobus (5 adult males, 35 adult or subadult females) from three groups in and around the edges of Jozani‐Chwaka Bay National Park, Zanzibar (6.233°S, 39.404°E). The Zanzibar red colobus is endemic to the island of Unguja where there are ca. 6000 individuals, 50% of which are found at this national park (Davenport et al., 2019). The subjects of this study are exposed to high levels of habitat disturbance and human activity from roads, tourism, and nearby villages and farms (Georgiev et al., 2019; Olgun et al., 2021; Siex & Struhsaker, 1999). They do not receive provisioned food. The mean maximum and minimum temperature between February 2019 and February 2020 at Jozani‐Chwaka Bay National Park were 34.5°C and 21.7°C, respectively (Zanzibar Red Colobus Project, unpublished data). The mean daily temperature variation was 12.7°C.

### 2.2 Urine sampling and storage

We opportunistically collected 225 urine samples from the 40 colobus (mean 6.5 samples per individual, range 1–29) typically between 7:00 and 18:00 h over a period of 12 months (August 2018–September 2019). We collected samples immediately after excretion from identified individuals which could be distinguished using facial markings and other distinguishing features (e.g., scars, injuries, and posture/shape). We either caught urine midstream using a plastic bag on the end of a catchpole or pipetted fresh urine splatter from the leaves. We only collected samples, which were not visibly contaminated with feces or detritus. Samples were carried in the dark and on ice in insulated lunchboxes until flash‐freezing in liquid nitrogen was possible later the same day. The samples were then transported to the UK in dry shippers below −150°C. Once at the laboratory, all samples were stored in −80°C freezers until assaying.

### 2.3 Freezing delay experiment

To examine the effect of varying delays to flash‐freezing of urine samples on OS marker measurement, we conducted a field experiment with seven urine samples. Upon collection of the sample, we briefly mixed and aliquoted each sample into four tubes and stored them on ice in an insulated lunchbox as described above. Upon return to the field base, we flash froze the first sample in liquid nitrogen (mean time since collection = 51 min, range 31–82 min), then the other aliquots were stored in the lunchbox until freezing at 2‐h intervals after the first [mean time between collection and freezing for the second aliquot was 169 min (range 151–202 min), 289 min for the third aliquot (range 271–322 min), and 413 min for the fourth aliquot (range 382–442 min)].

### 2.4 Oxidative stress marker analysis

We measured four markers of OS in all urine samples: a marker of DNA oxidative damage (8‐OHdG), a marker of lipid oxidative damage (MDA), and two markers of antioxidant capacity (TAC and UA). 8‐OHdG concentration was measured using the Invitrogen DNA Damage Competitive ELISA kit (catalogue number: EIADNAD). The concentration of MDA was measured using high‐performance liquid chromatography (HPLC) with no sample dilution. The concentration of TAC was measured using the Cayman Chemical Antioxidant Assay kit (catalog number: 709001). The concentration of UA was measured using the Cayman Chemical Uric Acid Assay kit (catalog number: 700320). All assays were carried out as per the manufacturer's instructions and to obtain values within the sensitivity range of the assay, we diluted samples 1:150 for 8‐OHdG, 1:100 for TAC, and 1:200 or 1:400 for UA. We adjusted the concentrations of all markers for urine dilution using specific gravity measured in the undiluted samples (Anestis et al., 2009). For 8‐OHdG, TAC, and UA, we assayed each sample in duplicate within the same plate and we repeated two samples across all plates as inter‐assay controls. The intra‐assay coefficients of variation (CVs) were 9.8% for 8‐OHdG, 7.2% for TAC, and 4.5% for UA. The inter‐assay coefficients of variation (CVs) were 11.7% for 8‐OHdG, 24.7% for TAC, and 14.8% for UA. Due to the high inter‐assay CV for TAC, we included plate as a random effect in the mixed model. To estimate the repeatability of measurement of HPLC, we analyzed 20 samples in duplicate which had an average CV of 4.22%.

We removed all samples for which specific gravity could not be measured due to values falling outside the detection range of the specific gravity meter (range 1–1.05) (*N* = 56). Two samples were removed because they had a low specific gravity (<1.004), which was leading to inflated marker concentrations (Thompson González et al., 2020). We removed 43 8‐OHdG measurements, 1 MDA measurement, 11 TAC measurements, and 1 UA measurement due to having a CV > 15%. Some samples were assayed for some markers and not others due to small sample volume and budgetary constraints.

### 2.5 Statistical analyses

#### 2.5.1 Cross‐sectional analysis: Testing the effects of environmental contamination, time of day, and duration of sample storage before and after freezing on OS marker measurement

We used a systematic model selection method in which we constructed a set of candidate GLM models to investigate the impact of the four methodological confounds [method of collection (two levels: plastic (*n* = 135) and leaves (*n* = 32)), time of day (decimal hours past midnight), freezing delay (decimal hours), and time‐frozen (decimal weeks)] on each OS marker. Because we did not have a priori predictions about the effects, each set of candidate models consisted of all combinations of the covariates and a null model containing only the random effects. Collection method was not included in the TAC and UA models because all assayed samples were collected on plastic. In all models, individual monkey ID was included as a random effect to account for multiple sampling of individuals. Plate was also included as a random effect in the TAC models due to these assays having high inter‐plate CVs. Finally, the number of freeze–thaw cycles was included as a fixed effect in the MDA model because 10 samples had 2 additional freeze–thaw cycles. This was included as a fixed effect instead of a random effect because it had only two levels. The number of freeze–thaw cycles of samples assayed for the remaining three markers was the same so it was not necessary to account for it statistically. For a full list of model structures, please see Table A1. The models were constructed using the lme4 package in R (Bates et al., 2015). We checked model residuals for normality and homogeneity by visual inspection of qqplots and scatterplots of fitted values versus standardized residuals, respectively. We log‐transformed 8‐OHdG, MDA, and UA measurements to homogenize and normalize the residuals. Collinearity was not an issue in these models (variance inflation factors <3.0; *car* package, Fox & Weisberg, 2019). The candidate models in each set were ranked based on AIC_c_ (Akaike's information criterion corrected for small sample size bias) to select the most parsimonious model with the lowest AIC_c_ value and highest AIC_c_ model weight. In each set of candidate models, more than one model had support (Δ[Q]AICc <2) and therefore we carried out multi‐model inference using model averaging with shrinkage in the AICcmodavg package in R (Mazerolle, 2020). Model averaging with shrinkage calculates weighted averages of the estimates based on all candidate models whereby models not containing the variable of interest are assigned a value of 0 for the β and variance. This is considered more robust method of model averaging than only averaging the models containing the variable of interest (Burnham & Anderson, 2002; Cade, 2015).

#### 2.5.2 Diurnal variation in OS markers

In addition to examining the effect of time of day on OS marker levels cross‐sectionally as described above, we identified matched pairs of samples that were collected in the morning (before 12 p.m., mean = 09:24 a.m.) and the afternoon (after 12 p.m., mean = 2:57 p.m.) from the same individual on the same day (nine pairs). If there were multiple samples in the morning or afternoon from the same individual, we averaged the marker concentrations across these samples. The average difference in collection time between morning and afternoon samples was 5 h and 36 min. We tested for differences between morning and afternoon urinary MDA levels using a Wilcoxon matched‐pairs test. This analysis was only carried out for MDA because there were not enough measurements of the other markers to conduct a matched‐pairs analysis following the exclusion of samples with an intra‐sample CV above 15%. We also calculated the coefficient of variation each for samples collected in the morning and for samples collected in the afternoon across the 12‐month dataset for all markers. We compared these CV values to determine whether OS markers are more variable in the morning or afternoon. These analyses were conducted on samples collected on plastic only.

#### 2.5.3 Field experiment testing the effect of freezing delay on OS marker levels

To investigate how consistent OS measurements were across 2‐h freezing delay increments, we calculated Kendall's concordance coefficients for each marker to investigate changes in rank order of samples following different freezing delays and we calculated the intra‐class correlation coefficient (ICC) for each marker based on single‐rating, absolute agreement, two‐way mixed‐effects model. These analyses were carried out using *DescTools* (Signorell et al., 2021) and *irr* (Gamer et al., 2019), respectively. To interpret the results of the ICC, we used commonly accepted cut‐off values for qualitative ratings of agreement where less than 0.5 = poor reliability, 0.5–0.75 = moderate reliability, 0.75–0.9 = good reliability, and 0.9 and above = excellent reliability (Koo & Li, 2016). Additionally, for illustrative purposes, we calculated the percentage change in OS concentration from time 0 for each freezing delay interval for each sample. All these samples were collected on plastic. This analysis was only conducted for MDA and 8‐OHdG due to budgetary constraints. After excluding samples as described above, we analyzed seven sets of aliquots for MDA and four for 8‐OHdG because three of the sets of 8‐OHdG aliquots were incomplete.

All analyses were carried out using R Studio (RStudio Team, 2020).

## 3 RESULTS

The final analytic dataset consisted of 108 8‐OHdG measurements, 167 MDA measurements, 100 TAC measurements, and 103 UA measurements collected between 07:15 a.m. and 5:54 p.m. over 47 non‐consecutive days. The freezing delay between sample collection and storage in liquid nitrogen ranged from 5 to 520 min (mean ± SE = 108 ± 6.7 min). Samples were kept frozen for a mean ± SE of 154 ± 9 days for 8‐OHdG (range = 64–440 days), 131 ± 9 days for MDA (range = 17–401 days), 761 ± 11 days for TAC (range = 664–1039 days), and 772 ± 12 days for UA (range = 667–1044 days) between collection in the field and assaying in the laboratory.

### 3.1 Cross‐sectional analysis: Testing the effects of environmental contamination, time of day, and duration of sample storage before and after freezing on OS marker measurement

Model selection identified 13, 11, 7, and 7 plausible models (Δ[Q]AICc <2) for 8‐OHdG, MDA, TAC, and UA, respectively (Appendix 1). Multi‐model averaging with shrinkage showed OS marker concentration was not affected by any of the investigated methodological confounds (Table 1).

**TABLE 1:** The model‐averaged estimates and confidence intervals with shrinkage for each model parameter

	Model‐averaged estimate with shrinkage	Unconditional SE	95% unconditional confidence interval
log(8‐OHdG [ng/ml corr. SG]) *N* = 108			
Method of collection	−0.16	0.12	−0.4, 0.08
Length of time frozen	0	0	0,0
Time of day	−0.01	0.01	−0.03, 0.02
Freezing delay	0.01	0.02	−0.03, 0.05
log(MDA [μM corr. SG]) *N* = 167			
Method of collection	−0.03	0.06	−0.16, 0.09
Length of time frozen	0	0	0, 0.01
Time of day	−0.02	0.01	−0.04, 0.01
Freezing delay	0	0.01	−0.02, 0.02
TAC (mM corr. SG) *N* = 100			
Length of time frozen	0.09	0.07	−0.04, 0.23
Time of day	−0.23	0.28	−0.78, 0.33
Freezing delay	0.11	0.33	−0.54, 0.77
log(UA [μM corr. SG]) *N* = 103			
Length of time frozen	0	0	0, 0.01
Time of day	−0.01	0.02	−0.04, 0.02
Freezing delay	0.01	0.02	−0.03, 0.04

### 3.2 Diurnal changes in OS

**FIGURE 1:**
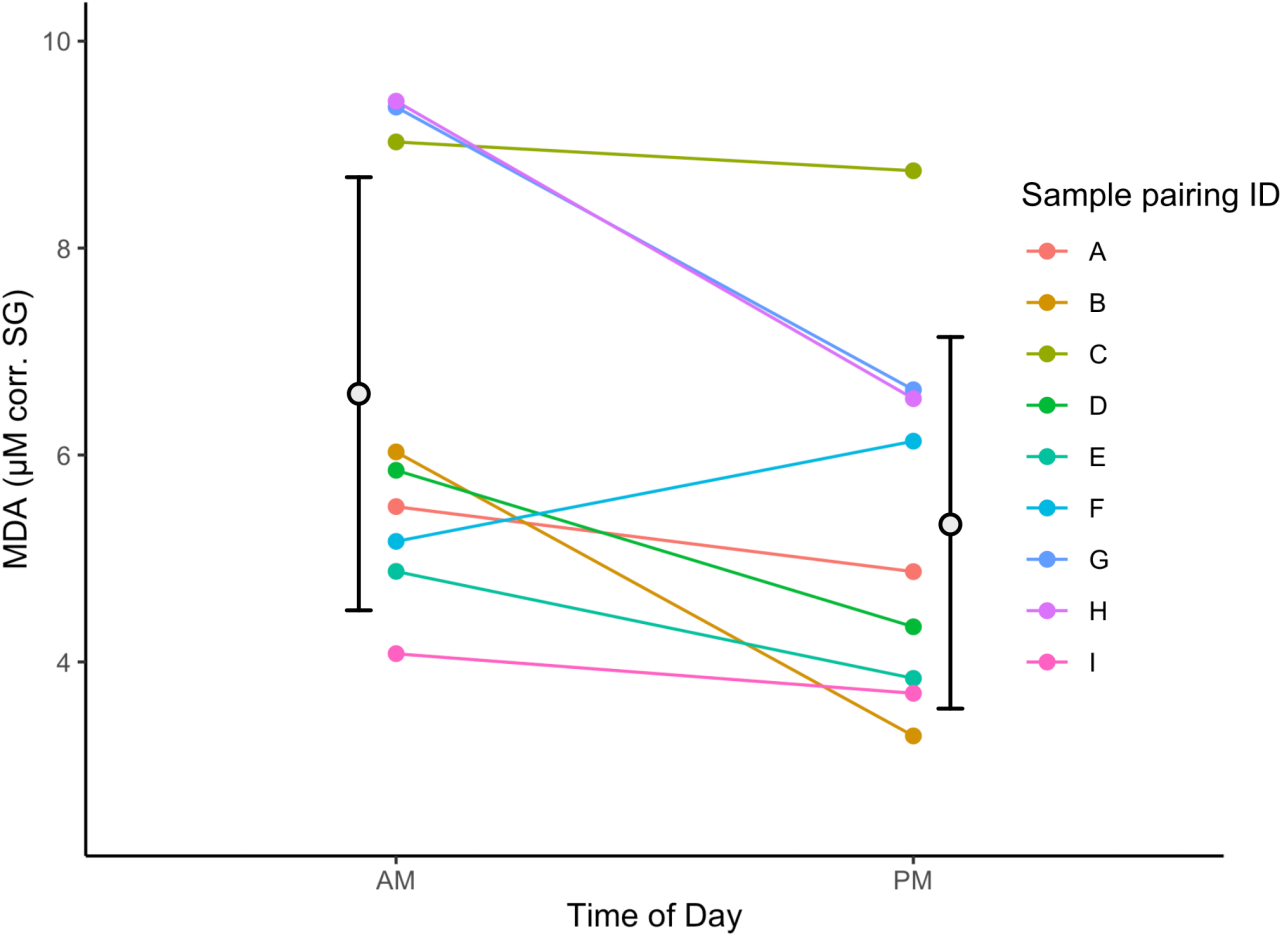
Difference in MDA concentration between pairs of samples collected from the same individual on the same day in the morning (before midday) and in the afternoon (after midday). The black open circles and lines represent the mean MDA concentration and standard deviation for morning and afternoon samples

MDA concentrations were higher in samples collected in the morning than in the afternoon (*N* = 9, V = 41, *p*‐value = .02734, Figure 1). Across the entire dataset (morning *N* = 78, afternoon *N* = 57), 8‐OHdG was more variable in samples collected in the morning (morning: CV = 45.9%, afternoon: CV = 36.9%), whereas MDA was more variable in samples collected in the afternoon (morning: CV = 44.2%, afternoon: CV = 54.2%). Marker concentrations were equally variable in the morning and the afternoon for both TAC and UA (TAC: morning: CV = 33.9%, afternoon: CV = 29.3%; UA: morning: CV = 40.9%, afternoon: CV = 43.2%).

### 3.3 Experimental test of the effect of freezing delay on OS

**FIGURE 2:**
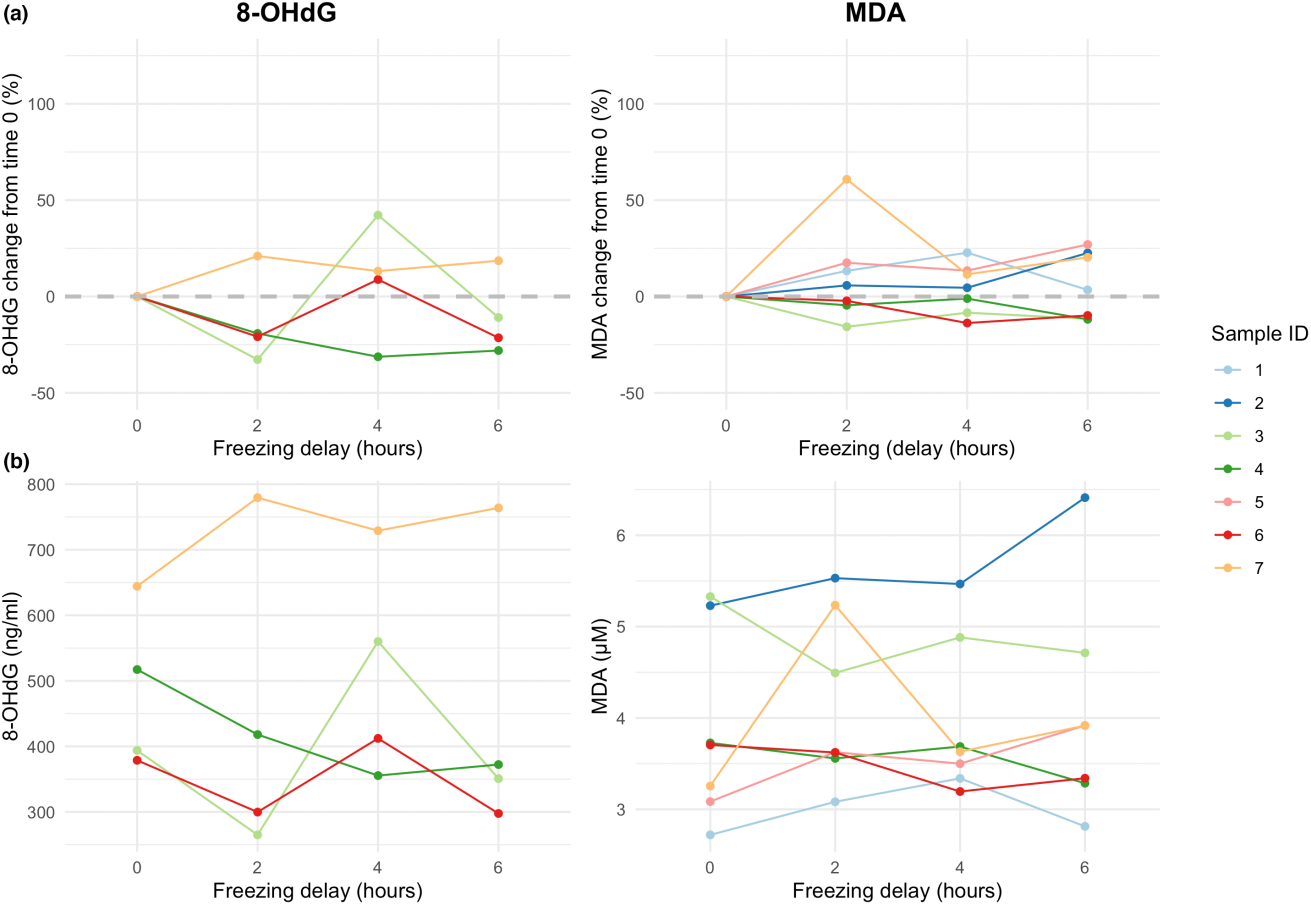
Effect of freezing delay on OS marker measurement (8‐OHdG and MDA, both corrected for specific gravity): (a) percentage change relative to values of time 0 controls; and (b) absolute value change. Controls were frozen as soon as possible after collection and then aliquots were frozen following 2‐, 4‐, and 6‐h delays. Sample numbers 1, 2, and 5 are missing in for 8‐OHdG due to failed measurements in some aliquots

Across the four freezing delay increments, 8‐OHdG levels were more variable than MDA levels (8‐OHdG percentage change: median = 20.9%, minimum = 8.8%, maximum = 42.2%; MDA percentage change: median = 11.5%, minimum = 1.1%, maximum = 60.8%), with MDA only exceeding ±30% for one measurement (Figure 2a). Similarly, the mean CV across freezing delays was 18.5% for 8‐OHdG (four samples) and 9.9% for MDA (seven samples). The Kendall's coefficients of concordance (W) between successive freezing delays were significant (8‐OHdG: W = 0.7, chi = 8.4, df = 3, *p* = .038; MDA: W = 0.76, chi = 18.21, df = 6, *p* = .006) and indicate moderate levels of concordance. The intraclass correlation coefficients demonstrate good reliability for 8‐OHdG measurements and good reliability for MDA measurements across freezing delay steps (sensu Koo & Li, 2016) (Table 2). Despite having moderate‐to‐good reliability between freezing delay steps, there was frequent rank‐order changes between the freezing delay steps (Figure 2b).

**TABLE 2:** Results of intraclass correlation coefficient (ICC) calculation using a single‐rating, absolute agreement, two‐way mixed‐effects model

			95% confidence interval		*F* test	
	*N*	ICC	Lower bound	Upper bound	Value	Significance
8‐OHdG	4	0.81	0.41	0.98	16	< 0.001
MDA	7	0.80	0.52	0.96	15.8	< 0.001

## 4 DISCUSSION

In this study, we demonstrate that OS markers are generally robust to methodological confounds that are common in field research. However, the reliability of some OS estimates may be affected by the duration of sample storage before freezing and the time of day the sample was collected. We highlight some potential confounds that future studies should consider and provide practical recommendations for the measurement of OS in urine collected non‐invasively and opportunistically from wild animals. Finally, we discuss the potential for use of urinary OS markers in field research.

### 4.1 Diurnal variation

While 8‐OHdG, TAC, and UA did not exhibit a pronounced diurnal variation in Zanzibar red colobus urine samples, MDA was higher in the morning than in the afternoon. This result was only apparent in the matched‐pairs analysis and not in the cross‐sectional analysis. Following the removal of samples with intra‐sample CVs above 15%, we did not have enough OS concentration measurements to conduct a matched‐pairs test for 8‐OHdG, TAC, and UA. Therefore, diurnal patterns could be present in these three markers if a matched‐pairs analysis were conducted in the future. Evidence for diurnal patterns in OS markers is mixed, with some studies showing no diurnal variation (urine: *Homo sapiens*, Grew et al., 2014; blood: *Fregata magnificens*, Sebastiano et al., 2017; and *Acinonyx jubatus*, Costantini et al., 2017) and some showing varying diurnal patterns even for the same markers (blood: *Homo sapiens*, Valencia et al., 2001; Kanabrocki et al., 2002; Singh et al., 2004; saliva: *Homo sapiens*, Alajbeg et al., 2017; Watanabe et al., 2019; urine: *Homo sapiens*, Kanabrocki et al., 2002; Miwa et al., 2004; and *Pan troglodytes*, Thompson González et al., 2020). One could argue that these conflicting conclusions may be due to the analytical procedures used. Where the analysis has been conducted in urine, variation in urine concentration could mask true diurnal patterns in OS production because urine concentration varies widely throughout the day. Some studies adjust OS measurements for urine concentration using specific gravity or creatinine (Thompson González et al., 2020) while others do not (Kanabrocki et al., 2002). We adjusted for urine concentration using specific gravity in this study, but still found conflicting results to those of Thompson González et al. (2020), who demonstrated, in wild chimpanzees, that MDA increased, and TAC decreased throughout the day. Therefore, the lack of a consistent diurnal pattern in OS markers in this study and others suggests that there may be a third variable driving OS marker concentrations that is associated with circadian rhythms but varies among sites, species, and sample sets within the same study system. Based on this, we suggest that urinary OS measurements should always be adjusted for urine concentration and each dataset should be examined for diurnal patterns as a precaution. If possible, researchers should collect urine samples consistently at one time of day unless the study design dictates otherwise.

### 4.2 Environmental contamination

We found that contact with leaves had no effect on OS measurements for any of the markers we studied. This lack of effect of leaf contamination is similar to that which has been observed in urinary steroid measurements (Knott, 2005; Marshall & Hohmann, 2005; Muller & Wrangham, 2004). This result suggests that urinary OS biomarkers are sufficiently stable to be collected from either plastic or leaf surfaces, which would give flexibility in sample collection method to field biologists that may not always succeed in placing a plastic sheet underneath their study subjects with full precision and perfect timing. We would, however, recommend additional testing of the impact of collection method on urinary OS marker concentration using an experimental approach following Muller and Wrangham (2004) and Knott (2005).

### 4.3 Storage time before freezing

Our cross‐sectional analysis showed that freezing delay up to 520 min did not have a significant effect on any of the four OS markers that we measured. Our study agrees with previous field research cross‐sectionally testing the effect of freezing delay on blood OS marker concentrations (Costantini et al., 2017; Nussey et al., 2009). However, our experimental analysis of MDA and 8‐OHdG measurements showed that values of these markers were highly variable and inconsistent across freezing delays of up to 360 min, more than has been observed in clinical OS studies (Lee & Kang, 2008; Matsumoto et al., 2008) and other urinary biomarkers (e.g., urinary neopterin: Heistermann & Higham, 2015). This discrepancy might be due to a difference in ambient temperature at our tropical field site and in the lab conditions used in these studies. The difference in results between our cross‐sectional and experimental study suggests that freezing delay may not be an issue for higher‐level comparisons of OS between groups of samples, but that comparisons between individual samples exposed to different freezing delays may not be informative. An investigation of the rank orders of individual samples is missing from the previous studies of freezing delay and urinary OS measures (Lee & Kang, 2008; Matsumoto et al., 2008) and therefore these fine‐scale differences between samples may have been overlooked. Future research should consider the potential confounding effects of storage on OS measurements, especially if individual samples are to be compared.

We found that for both 8‐OHdG and MDA, OS concentration oscillated across freezing delay steps where we would expect a steady decrease. Even though samples were mixed using a pipette prior to aliquoting, we suspect this oscillation might be caused by drawing the four aliquots from a heterogenous urine sample, rather than due to lab measurement error, as that was low (CV <15%). Therefore, there may be additional confounding factors affecting the reliable comparison of OS measurements from individual samples and we advise that spot sampling (whereby single samples are taken to represent the concentration for a unit of analysis, e.g., individuals, sites, and periods) should be avoided. Instead, multiple samples should be taken to calculate mean OS concentrations for each unit of analysis.

MDA was more stable across the four freezing delay steps than 8‐OHdG. In fact, using a well‐accepted CV cut‐off value of 15%, most of the 8‐OHdG measurements would be deemed unreliable if the aliquots were treated as measurement replicates. There is no indication in the literature that 8‐OHdG is a less stable molecule than MDA (Cooke et al., 2008) so the difference in variability between the markers most likely comes from the methods we used to quantify them (ELISA for 8‐OHdG and HPLC for MDA). While ELISAs have been widely used to measure 8‐OHdG in urine samples, they are subject to greater measurement variability (Barregard et al., 2013) than chromatographic approaches such as HPLC (Graille et al., 2020). An ELISA is adequate for comparing relative urinary OS levels between groups (where multiple samples contribute to a mean estimate) but not to reliably measure exact concentrations of markers (Cooke et al., 2008; Yoshida et al., 2002). Researchers should be aware of the analytical limitations of different methods and select the most appropriate method to address their question. For example, if individual samples need to be compared, HPLC approaches, which have higher specificity and sensitivity, would be more desirable.

### 4.4 Sample storage after freezing

In our correlational analysis, all OS marker measurements remained stable across frozen storage times, which is supported by previous research demonstrating high levels of stability of various OS markers during storage below −20°C for over 1 year (blood: Koracevic et al., 2001; Jansen et al., 2013b; Costantini et al., 2017; Jansen et al., 2017; Rubio et al., 2018; urine: Matsumoto et al., 2008; Martinez & Kannan, 2018; Thompson González et al., 2020). Our result must be caveated with the fact that we only tested the effect of duration of storage at −80°C up to 15 months for MDA and 8‐OHdG, and for up to 35 months for TAC and UA. Other studies have demonstrated degradation of OS markers over different time periods and at different temperatures. For example, Martinez and Kannan (2018) found a 40% decrease in MDA in human urine samples after 30 days of storage at −20°C and Thompson González et al. (2020) demonstrated a weak negative effect of storage time (1–10 years) at −30°C on MDA‐TBARS in urine from wild chimpanzees. Therefore, we still recommend that samples should, ideally, be stored at −80°C and for as short a time as is possible, and that the potential effect of time in storage on marker values should be examined for each dataset. However, our result is promising for future studies utilizing OS markers, both in field and laboratory environments.

## 5 CONCLUSIONS

The application of non‐invasive OS measurement in the field will strengthen our ability to address exciting theoretical and applied questions, for example, the role of OS in life‐history trade‐offs, development, aging, reproduction, and the effects of anthropogenic disturbance and environmental conditions on wild animal physiology, health, and fitness. The redox system is highly conserved across taxa and plays an important role in many biological processes. Therefore, it can be studied in a wide variety of contexts and study systems, something that will only be aided by being able to study natural populations in a non‐invasive way. Additionally, being able to investigate these questions in populations engaging in natural social interactions and facing natural resource restrictions will make the insights more pertinent than those that might be gained through laboratory studies.

However, applying physiological methods in the field increases the potential for methodological confounds (e.g., from environmental contamination, differences in freezing delays, heterogeneous urine samples, or assay method). Here, we demonstrated a high level of stability of four urinary OS markers in response to four common methodological confounds of field research. In general, we advise that future studies should be mindful of the potential for diurnal patterns in OS markers and of the potential confounding effects of storage on OS measurements. In particular, we advise that markers of OS concentrations should be adjusted for urine concentration, samples should be collected at one time of day, and samples should be stored at −80°C for the shortest time possible. Additionally, researchers should avoid spot sampling to ensure the reliability of their results. Nevertheless, our results provide encouraging evidence that these markers are sufficiently stable to conduct a robust study of OS in non‐invasively collected urine samples from wild animals within reasonable methodological constraints.

## AUTHOR CONTRIBUTIONS

**Zoe Elizabeth Melvin:** Conceptualization (equal); data curation (lead); formal analysis (lead); funding acquisition (equal); investigation (lead); methodology (lead); project administration (equal); visualization (lead); writing – original draft (lead); writing – review and editing (lead). **Hussein Dhirani:** Data curation (supporting); investigation (supporting); project administration (supporting); writing – review and editing (supporting). **Christopher Mitchell:** Investigation (supporting); methodology (supporting); project administration (supporting); resources (supporting); supervision (supporting); writing – review and editing (supporting). **Tim Davenport:** Conceptualization (equal); funding acquisition (equal); methodology (equal); project administration (supporting); resources (supporting); supervision (equal); writing – review and editing (equal). **Jonathan Blount:** Conceptualization (equal); formal analysis (equal); funding acquisition (equal); investigation (equal); methodology (equal); project administration (supporting); resources (equal); supervision (equal); writing – review and editing (equal). **Alexander Georgiev:** Conceptualization (equal); data curation (supporting); formal analysis (equal); funding acquisition (equal); investigation (equal); methodology (equal); project administration (equal); resources (equal); supervision (lead); visualization (supporting); writing – original draft (supporting); writing – review and editing (equal).

## CONFLICT OF INTEREST

All authors gave final approval for publication and have no conflicts of interest.

## Data Availability

Data have been shared on Dryad, https://doi.org/10.5061/dryad.c2fqz61bx.

## APPENDIX A

**TABLE A1:** AICc tables used for model selection and multi‐model inference for the candidate set of models for each OS marker and the subsequent estimates and standard errors for each model parameter

(A) Log(8‐OHdG [ng/ml corr. SG])					Method		Frozen storage		Time		Freezing D	
Model structure	K	AICc	ΔAICc	AICcWt	Estimate	SE	Estimate	SE	Estimate	SE	Estimate	SE
Method	**4**	**96.22**	**0**	**0.19**	−0.21	0.1	0	0	0	0	0	0
Method + Time	**5**	**96.48**	**0.26**	**0.16**	−0.2	0.1	0	0	−0.02	0.01	0	0
Method + Freezing D	**5**	**96.85**	**0.63**	**0.14**	−0.22	0.1	0	0	0	0	0.03	0.02
Method + Time + Freezing D	**6**	**97.64**	**1.42**	**0.09**	−0.21	0.1	0	0	−0.02	0.01	0.03	0.02
Method + Frozen Storage	**5**	**98.42**	**2.2**	**0.06**	−0.21	0.1	0	0	0	0	0	0
Time	**4**	**98.69**	**2.47**	**0.05**	0	0	0	0	−0.02	0.01	0	0
Null	**3**	**98.7**	**2.49**	**0.05**	0	0	0	0	0	0	0	0
Method + Frozen Storage + Time	**6**	**98.72**	**2.5**	**0.05**	−0.2	0.1	0	0	−0.02	0.01	0	0
Method + Frozen Storage + Freezing D	**6**	**99.07**	**2.85**	**0.05**	−0.22	0.1	0	0	0	0	0.03	0.02
Method + Frozen Storage + Time + Freezing D	**7**	**99.89**	**3.68**	**0.03**	−0.21	0.1	0	0	−0.02	0.01	0.03	0.02
Freezing D	**4**	**99.93**	**3.71**	**0.03**	0	0	0	0	0	0	0.02	0.02
Time + Freezing D	**5**	**100.34**	**4.12**	**0.02**	0	0	0	0	−0.02	0.01	0.02	0.02
Frozen Storage + Time	**5**	**100.57**	**4.35**	**0.02**	0	0	0	0	−0.02	0.01	0	0
Frozen Storage	4	100.59	4.38	0.02	0	0	0	0	0	0	0	0
Frozen storage + Freezing D	5	101.65	5.43	0.01	0	0	0	0	0	0	0.03	0.02
Frozen Storage + Time + Freezing D	6	102.08	5.87	0.01	0	0	0	0	−0.02	0.01	0.02	0.02

(B) Log(MDA [μM corr. SG])					Method		Frozen storage		Time		Freezing D	
Model structure	K	AICc	ΔAICc	AICcWt	Estimate	SE	Estimate	SE	Estimate	SE	Estimate	SE
Frozen Storage + Time	**6**	**162.52**	**0**	**0.27**	0	0	0.01	0	−0.02	0.01	0	0
Method + Frozen Storage + Time	**7**	**163.53**	**1.01**	**0.16**	−0.08	0.07	0	0	−0.02	0.01	0	0
Frozen Storage + Time + Freezing D	**7**	**164.6**	**2.08**	**0.1**	0	0	0.01	0	−0.02	0.01	−0.01	0.02
Frozen Storage	**5**	**165.33**	**2.82**	**0.07**	0	0	0.01	0	0	0	0	0
Method + Frozen Storage + Time + Freezing D	**8**	**165.7**	**3.18**	**0.06**	−0.08	0.07	0	0	−0.02	0.01	0	0.02
Method + Frozen Storage	**6**	**166.22**	**3.7**	**0.04**	−0.08	0.08	0	0	0	0	0	0
Method + Time	**6**	**166.81**	**4.29**	**0.03**	−0.11	0.07	0	0	−0.02	0.01	0	0
Time	**5**	**166.99**	**4.47**	**0.03**	0	0	0	0	−0.02	0.01	0	0
Null	**3**	**167.43**	**4.91**	**0.02**	0	0	0	0	0	0	0	0
Frozen Storage + Freezing D	**6**	**167.49**	**4.97**	**0.02**	0	0	0.01	0	0	0	0	0.02
Method + Frozen Storage + Freezing D	**7**	**168.37**	**5.85**	**0.01**	−0.09	0.08	0	0	0	0	0	0.02
Time + Freezing D	6	168.88	6.36	0.01	0	0	0	0	−0.02	0.01	−0.01	0.02
Method + Time + Freezing D	7	168.91	6.39	0.01	−0.11	0.07	0	0	−0.02	0.01	−0.01	0.02
Method	5	169.16	6.64	0.01	−0.12	0.07	0	0	0	0	0	0
Method + Freezing D	6	171.31	8.79	0	−0.12	0.08	0	0	0	0	0	0.02
Freezing D	5	171.54	9.02	0	0	0	0	0	0	0	0	0.02

(C) TAC (mM corr. SG)					Frozen storage		Time		Freezing D	
Model structure	K	AICc	ΔAICc	AICcWt	Estimate	SE	Estimate	SE	Estimate	SE
Frozen Storage + Time	**6**	**697.25**	**0**	**0.31**	0.12	0.06	−0.43	0.25	0	0
Frozen Storage	**5**	**697.84**	**0.59**	**0.23**	0.12	0.06	0	0	0	0
Frozen Storage + Time + Freezing D	**7**	**699.24**	**1.98**	**0.12**	0.12	0.06	−0.4	0.26	0.3	0.52
Frozen Storage + Freezing D	**6**	**699.31**	**2.06**	**0.11**	0.12	0.06	0	0	0.46	0.52
Time	**5**	**699.94**	**2.68**	**0.08**	0	0	−0.4	0.26	0	0
Null	**4**	**700.09**	**2.84**	**0.08**	0	0	0	0	0	0
Freezing D	**5**	**701.61**	**4.36**	**0.04**	0	0	0	0	0.44	0.53
Time + Freezing D	6	701.9	4.65	0.03	0	0	−0.38	0.27	0.29	0.53

(D) Log(UA [μM corr. SG])					Frozen storage		Time		Freezing D	
Model structure	K	AICc	ΔAICc	AICcWt	Estimate	SE	Estimate	SE	Estimate	SE
Frozen Storage + Time	**5**	**110.19**	**0**	**0.28**	**0.01**	**0**	**−0.02**	**0.01**	**0**	**0**
Frozen Storage	**4**	**110.45**	**0.26**	**0.24**	**0.01**	**0**	**0**	**0**	**0**	**0**
Frozen Storage + Freezing D	**5**	**111.72**	**1.53**	**0.13**	**0.01**	**0**	**0**	**0**	**0.03**	**0.03**
Frozen Storage + Time + Freezing D	**6**	**111.85**	**1.66**	**0.12**	**0.01**	**0**	**−0.02**	**0.01**	**0.02**	**0.03**
Time	**4**	**112.46**	**2.27**	**0.09**	**0**	**0**	**−0.02**	**0.01**	**0**	**0**
Null	**3**	**112.87**	**2.68**	**0.07**	**0**	**0**	**0**	**0**	**0**	**0**
Time + Freezing D	**5**	**114.25**	**4.06**	**0.04**	**0**	**0**	**−0.02**	**0.01**	**0.02**	**0.03**
Freezing D	4	114.31	4.12	0.04	0	0	0	0	0.02	0.03

*Note*: Method = collection method (plastic or leaves), Freezing D = freezing delay (decimal hours), Time = time of sample collection (decimal hours past midnight), and Frozen Storage = length of time in frozen storage (decimal weeks). The null model included just the random effects. K = number of parameters, AIC_c_ = the second‐order Akaike's information criterion, ΔAICc = the difference between the AICc of each model and the top‐ranked model, and AICcWt is the AIC weight of each model. Estimates and SE are set to 0 for models that do not contain the variable of interest so as not to inflate estimate values. The models in bold represent the 95% confidence set of models.
